# Obstructive Sleep Apnea and Venous Thromboembolism: Unraveling the Emerging Association

**DOI:** 10.7759/cureus.44367

**Published:** 2023-08-30

**Authors:** Sahil P Bhutada, Ishwar Agrawal, Ajinklya Punpale, Viresh Kannure, Roshan Prasad, Tejaswee Lohakare, Mayur Wanjari, Gaurav Mittal

**Affiliations:** 1 Medicine, Jawaharlal Nehru Medical College, Datta Meghe Institute of Higher Education and Research, Wardha, IND; 2 Surgical Oncology, Jawaharlal Nehru Medical College, Datta Meghe Institute of Higher Education and Research, Wardha, IND; 3 Internal Medicine, Jawaharlal Nehru Medical College, Datta Meghe Institute of Higher Education and Research, Wardha, IND; 4 Child Health Nursing, Srimati Radhikabai Meghe Memorial College of Nursing, Wardha, IND; 5 Research and Development, Jawaharlal Nehru Medical College, Datta Meghe Institute of Higher Education and Research, Wardha, IND; 6 Sports Medicine, Mahatma Gandhi Institute of Medical Sciences, Wardha, IND

**Keywords:** emerging, management, clinical implications, mechanisms, association, venous thromboembolism, obstructive sleep apnea

## Abstract

Oxidative stress has emerged as a significant contributor to skeletal muscle atrophy, influencing cellular processes that underlie muscle wasting. This review article delves into the intricate interplay between oxidative stress and muscle atrophy, shedding light on its mechanisms and implications. We begin by outlining the fundamental concepts of oxidative stress, delineating reactive oxygen species (ROS) and reactive nitrogen species (RNS), their sources, and the ensuing oxidative damage to cellular components. Subsequently, we delve into skeletal muscle atrophy, elucidating its diverse forms, molecular pathways, key signaling cascades, and the role of inflammation in exacerbating muscle wasting. Bridging these concepts, we explore the connections between oxidative stress and muscle atrophy, unveiling how oxidative stress impacts muscle protein synthesis and breakdown, perturbs cellular signaling pathways, and contributes to mitochondrial dysfunction. The review underscores the complexity of quantifying and interpreting oxidative stress markers, highlighting the challenges posed by the dynamic nature of oxidative stress and the presence of basal ROS levels. Addressing the specificity of oxidative stress markers, we emphasize the importance of selecting markers pertinent to muscle tissue and considering systemic influences. Standardization of experimental protocols emerges as a critical need to ensure consistency and reproducibility across studies. Looking ahead, we discuss the implications of oxidative stress in diverse scenarios, encompassing age-related muscle loss (sarcopenia), muscle wasting in chronic diseases like cancer cachexia, and disuse-induced muscle atrophy. Additionally, we delve into potential therapeutic strategies, including antioxidant supplementation, exercise, pharmacological interventions, nutritional approaches, and lifestyle modifications, as avenues to mitigate oxidative stress-driven muscle atrophy. The review concludes by outlining promising future directions in this field, calling for deeper exploration of specific oxidative stress markers, understanding the temporal dynamics of oxidative stress, validation through translational studies in humans, and the development of targeted therapeutic interventions. By advancing our understanding of the intricate relationship between oxidative stress and skeletal muscle atrophy, this review contributes to paving the way for innovative strategies to address muscle wasting and improve muscle health.

## Introduction and background

Obstructive sleep apnea (OSA) and venous thromboembolism (VTE) are two prevalent and significant medical conditions that have traditionally been studied and managed independently. However, emerging evidence suggests a potential association between these seemingly unrelated disorders. OSA is a sleep-related breathing disorder characterized by recurrent episodes of partial or complete upper airway obstruction during sleep, leading to disrupted sleep patterns and intermittent hypoxia. On the other hand, VTE encompasses both deep vein thrombosis (DVT) and pulmonary embolism (PE) and occurs due to the formation of blood clots within the venous system [1-3].

OSA affects millions of individuals worldwide, with varying degrees of severity. It is often underdiagnosed, leading to substantial health and economic burdens [4]. In more recent population-based studies, the prevalence of OSA was estimated to be higher, from 14% to 50% in men and from 5% to 23% in women [4]. The primary pathophysiology of OSA involves the relaxation and collapse of the upper airway muscles during sleep, resulting in airflow cessation. This disruption in breathing patterns leads to brief awakenings and intermittent hypoxia, causing a cascade of physiological responses that can adversely affect cardiovascular health and overall well-being [4,5].

VTE, encompassing DVT and PE, constitutes a significant global health concern, posing substantial morbidity and mortality risks [6]. The formation of blood clots within the venous system, frequently occurring in the lower extremities, can culminate in severe complications unless timely diagnosis and intervention are initiated. Multiple factors converge to precipitate VTE, with recognized triggers encompassing immobility, surgical procedures, malignancy, and inherent hypercoagulable conditions [6,7]. Furthermore, it is crucial to acknowledge that the spectrum of risk factors extends beyond these well-established categories. Trauma and fractures, for instance, emerge as additional contributors with the potential to instigate VTE. This underscores the intricate nature of VTE etiology and the imperative of a comprehensive risk assessment approach.

While the pathophysiology of OSA and VTE may appear distinct at first glance, recent research has begun to shed light on a possible link between the two conditions. Studies have suggested that the intermittent hypoxia experienced by individuals with OSA might contribute to a prothrombotic state, increasing the risk of VTE development. Additionally, shared risk factors and underlying comorbidities between OSA and VTE could play a role in this emerging association [8].

This review article aims to comprehensively explore and evaluate the emerging association between OSA and VTE. By synthesizing the existing literature and recent research findings, we aim to better understand the potential interplay between these conditions. Furthermore, this review will highlight the possible mechanisms linking OSA and VTE, discuss relevant clinical studies investigating their association, and explore the clinical implications for patient management and care.

## Review

Methodology

**Figure 1:**
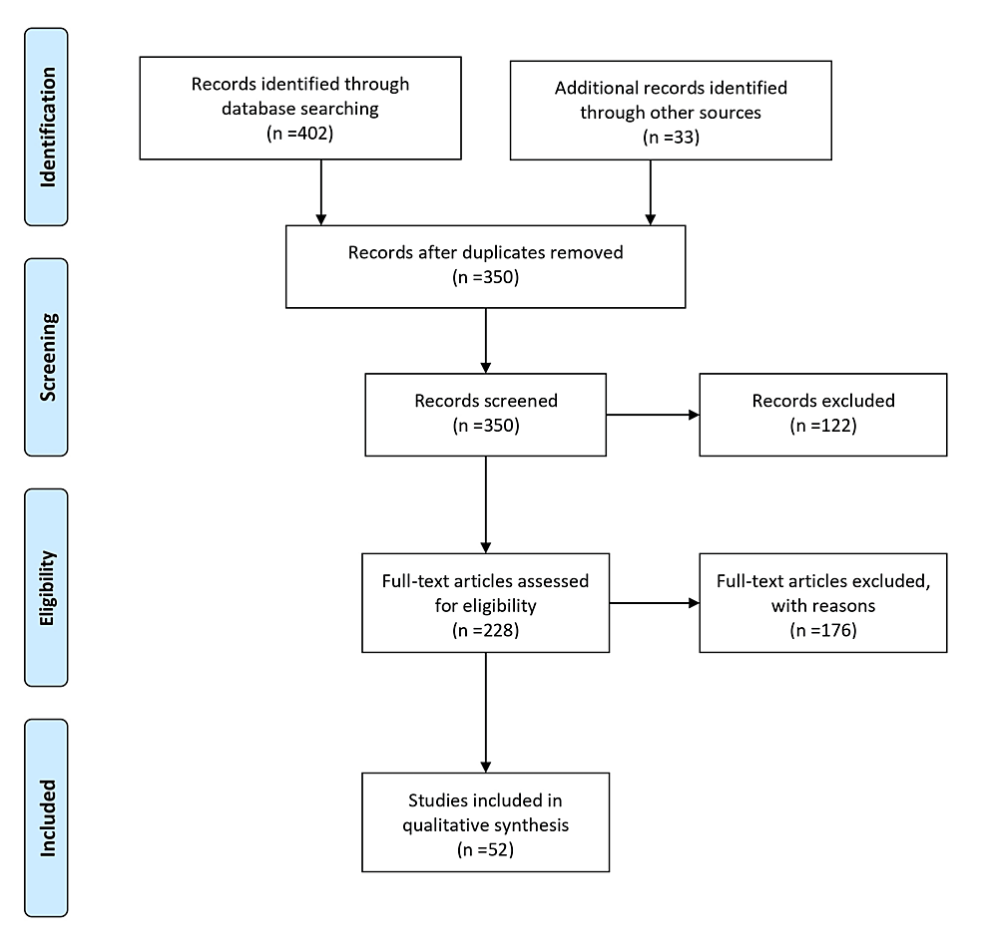
The selection process of articles used in this study. Adopted from the Preferred Reporting Items for Systematic Reviews and Meta-Analyses (PRISMA).

A literature search was conducted to comprehensively review the association between OSA and venous thromboembolism (VTE). Multiple electronic databases, including PubMed, MEDLINE, Embase, and Google Scholar, were searched using the following keywords and combinations: obstructive sleep apnea, sleep-disordered breathing, OSA, venous thromboembolism, deep vein thrombosis, and pulmonary embolism. The search encompassed articles published from 2000 to 2022. In addition to electronic database searches, reference lists of relevant articles and review papers were manually screened to identify additional studies. The inclusion criteria involved selecting observational studies, experimental studies, systematic reviews, and meta-analyses that examined the association between OSA and VTE and their impact on related outcomes. Studies with adult human participants were included, while those focusing solely on other sleep disorders or non-VTE thrombotic conditions were excluded. Only peer-reviewed, published articles were considered for inclusion. Two independent reviewers assessed the titles, abstracts, and full-text articles for eligibility, with any discrepancies resolved through discussion and consensus. The comprehensive literature search aimed to ensure the inclusion of relevant studies and provide a thorough analysis of the association between OSA and VTE. Figure 1 describes the selection process of articles used in our study.

Overview of VTE

Definition and Pathophysiology of VTE

VTE refers to the formation of blood clots within the venous system, leading to potentially life-threatening conditions such as DVT and PE. DVT occurs when a clot forms within a deep vein, most commonly in the lower extremities. If a portion of the clot dislodges and travels to the lungs, it can result in PE, which can be fatal [1].

The pathophysiology of VTE involves a combination of Virchow's triad: endothelial injury, hypercoagulability, and venous stasis. Endothelial injury, which can occur due to trauma, surgery, or inflammation, disrupts the normal anticoagulant properties of the blood vessels. Hypercoagulability is an increased tendency for blood to clot, which can arise from inherited or acquired conditions such as genetic mutations, cancer, hormonal changes, or certain medications. Venous stasis occurs when blood flow becomes sluggish or stagnant, often due to immobility, obesity, or venous insufficiency, creating a favorable environment for clot formation [4].

Prevalence and Risk Factors

VTE is a prevalent condition that affects millions of individuals worldwide. The incidence and prevalence of VTE vary depending on the population studied, with higher rates observed in older individuals and those with underlying comorbidities. VTE can occur in individuals of any age, including both sexes, although certain risk factors increase the likelihood of its development [9,10].

Common risk factors for VTE include immobility (e.g., prolonged bed rest, long-distance travel), surgery (especially orthopedic and abdominal procedures), trauma, malignancy, pregnancy and the postpartum period, hormonal therapy (such as oral contraceptives or hormone replacement therapy), inherited thrombophilia (such as Factor V Leiden mutation or prothrombin gene mutation), and prior history of VTE. Obesity, smoking, and certain medical conditions, such as heart failure or inflammatory disorders, can also contribute to an increased risk of VTE [11,12].

Clinical Presentation and Diagnosis

The clinical presentation of VTE can vary depending on the location and extent of the clot. In DVT, patients often present with pain, swelling, warmth, and erythema in the affected limb. However, some cases of DVT can be asymptomatic or present with atypical symptoms. In PE, common symptoms include sudden onset dyspnea, chest pain, cough, hemoptysis, and signs of hemodynamic instability [13].

The diagnosis of VTE involves a combination of clinical assessment, imaging studies, and laboratory tests. Imaging modalities such as compression ultrasound or venography are used to confirm the presence of DVT, while computed tomography pulmonary angiography (CTPA) or ventilation-perfusion (V/Q) scans are employed to diagnose PE. Laboratory tests, including D-dimer assay and specific coagulation tests, can help support the diagnosis and assess the probability of VTE [14].

Impact of VTE on Overall Health

VTE has significant implications for overall health and can lead to acute and chronic complications. Acute complications include the potential for PE, which can result in respiratory failure, hemodynamic instability, and even death. Chronic complications of VTE include post-thrombotic syndrome, characterized by chronic leg pain, swelling, and skin changes, which can significantly impair quality of life. Additionally, VTE recurrence can occur, especially in individuals with ongoing risk factors or underlying prothrombotic conditions [15,16].

VTE is also associated with long-term complications such as chronic thromboembolic pulmonary hypertension (CTEPH), characterized by persistent pulmonary hypertension due to organized thrombi within the pulmonary arteries. Furthermore, VTE has been linked to an increased risk of cardiovascular events, including heart attack and stroke [17].

Mechanisms linking OSA and VTE

Shared Risk Factors and Comorbidities

OSA and VTE exhibit an intriguing overlap in their risk factors and comorbidities, potentially contributing to the observed association between the two conditions. Notably, obesity stands out as a prominent shared risk factor. Excess adipose tissue in individuals with obesity can lead to chronic inflammation and an imbalance in the production of prothrombotic factors, thereby increasing the risk of both OSA and VTE. The visceral fat accumulation further exacerbates these effects, creating a vicious cycle of inflammation and thrombogenesis [2].

Advancing age is another common risk factor for both OSA and VTE. As individuals age, the prevalence of OSA and VTE tends to increase. Aging is associated with anatomical changes in the upper airway and alterations in coagulation pathways, which may predispose individuals to OSA-related airway collapse and VTE formation [18].

A sedentary lifestyle, often coupled with obesity, plays a dual role in developing and progressing OSA and VTE. The relationship between obesity and sedentary behavior is integral to understanding how weight gain precipitates OSA. Excessive weight gain associated with sedentary habits contributes to increased adiposity, particularly around the upper airway. This can lead to airway obstruction, a hallmark of OSA. Concurrently, sedentary living promotes muscle deconditioning and circulatory impairment, further contributing to the pathophysiology of OSA and VTE. Furthermore, sedentary behavior extends its influence beyond mechanical factors. It fosters a proinflammatory and prothrombotic environment, perpetuating the risk of both conditions. The lack of physical activity is intricately linked to inflammatory mediators' release and endothelial function disruption. These effects synergize with obesity to enhance the prothrombotic state, promoting the development of VTE [19].

Several medical conditions exhibit a notable association with both OSA and VTE, warranting an in-depth exploration of their shared pathophysiological mechanisms. Metabolic syndrome, a conglomerate of conditions encompassing obesity, hypertension, dyslipidemia, and insulin resistance, prominently manifests in individuals afflicted by OSA and VTE. This intricate interplay of metabolic disturbances significantly contributes to the genesis of endothelial dysfunction, chronic inflammation, and a heightened propensity for thrombotic events. Of particular significance, obesity-driven adipose tissue dysfunction amplifies the release of pro-inflammatory cytokines and adipokines, precipitating a state of chronic inflammation that sets the stage for the initiation and progression of thrombosis [20]. Additionally, the intricate connection between OSA, VTE, and diabetes mellitus further accentuates the prothrombotic milieu. Diabetes, marked by hyperglycemia and insulin resistance, augments the risk of both OSA and VTE. Hyperglycemia disrupts endothelial function, promoting a pro-inflammatory and pro-coagulant state, while insulin resistance accentuates oxidative stress and systemic inflammation. In tandem, these diabetes-associated alterations exacerbate the susceptibility to thrombosis, thus establishing a multifaceted linkage between these clinical entities [20].

Cardiovascular diseases, including hypertension, coronary artery disease, and congestive heart failure, frequently coexist in individuals grappling with OSA and VTE. Shared pathophysiological mechanisms intertwine these conditions, with OSA-mediated intermittent hypoxia fostering sympathetic nervous system activation and consequent hemodynamic fluctuations. This chronic interplay of hypoxia-reoxygenation cycles propels oxidative stress, inflammation, and endothelial dysfunction, which collectively fuel the progression of cardiovascular pathology. These intricate connections substantiate the propensity for VTE occurrence through the induction of prothrombotic milieu and endothelial dysfunction [20]. Taken collectively, the intricate relationships between OSA, VTE, and various comorbidities unveil a complex web of pathophysiological mechanisms. The confluence of metabolic perturbations, systemic inflammation, and cardiovascular dysregulation is a nexus for the interplay between these conditions, orchestrating a heightened prothrombotic state. Understanding these mechanistic intricacies is pivotal in delineating targeted therapeutic interventions and management strategies that hold the potential to mitigate the augmented thrombotic risk in individuals grappling with OSA and VTE [20].

Role of Intermittent Hypoxia and Oxidative Stress

The role of intermittent hypoxia and oxidative stress in the association between OSA and VTE is important. Intermittent hypoxia is a characteristic feature of OSA due to repeated episodes of partial or complete upper airway obstruction during sleep. During these episodes, oxygen levels in the blood decrease, leading to oxygen desaturation. Subsequently, when the airway obstruction is relieved, reoxygenation occurs [21].

The cycle of intermittent hypoxia and reoxygenation in OSA can harm the body. One such effect is the generation of oxidative stress. Intermittent hypoxia triggers the production of reactive oxygen species (ROS) in the body, leading to oxidative stress. ROS are highly reactive molecules that can cause damage to cells and tissues. They have been implicated in various pathological processes, including inflammation and endothelial dysfunction [22].

The oxidative stress induced by intermittent hypoxia in OSA has several consequences contributing to the association between OSA and VTE. Firstly, oxidative stress promotes inflammation, as ROS can activate inflammatory pathways. This chronic inflammation can lead to endothelial dysfunction, impairing the normal functioning of the endothelium, which lines the blood vessels. Endothelial dysfunction is associated with an imbalance in coagulation and fibrinolysis, favoring a pro-thrombotic state [23]. Furthermore, oxidative stress can directly affect clot formation and dissolution balance. ROS can promote platelet activation and aggregation and activate the coagulation cascade, increasing the risk of thrombus formation. Additionally, oxidative stress can impair the fibrinolytic system, which is responsible for the dissolution of blood clots, further contributing to a pro-thrombotic state.

Impact of Inflammation and Endothelial Dysfunction

Both OSA and VTE have been associated with systemic inflammation and endothelial dysfunction, highlighting potential shared pathways in their pathogenesis. OSA is known to induce chronic low-grade inflammation, characterized by increased production of pro-inflammatory cytokines and elevated levels of circulating inflammatory markers. This inflammatory milieu can disrupt the delicate balance of endothelial function, leading to impaired vasodilation, increased vasoconstriction, and a prothrombotic state. These alterations in the endothelium may contribute to the development of venous thrombosis and the pathogenesis of VTE [24].

The chronic inflammation observed in OSA is attributed to several mechanisms, including intermittent hypoxia, oxidative stress, and immune system activation. Intermittent hypoxia, a hallmark of OSA, triggers the release of pro-inflammatory mediators, stimulates the production of ROS, and activates inflammatory pathways. These processes can lead to endothelial cell dysfunction and the release of endothelial-derived factors that promote thrombosis [25].

Endothelial dysfunction, characterized by impaired nitric oxide bioavailability and increased expression of adhesion molecules, further contributes to the prothrombotic state in OSA and VTE. The dysregulated endothelium exhibits reduced vasodilatory capacity, increased pro-coagulant factor expression, and enhanced platelet adhesion and leukocyte adhesion to the endothelial surface. These changes disrupt the antithrombotic properties of the endothelium, favoring a prothrombotic phenotype and increasing the risk of VTE development [26].

Inflammation and endothelial dysfunction are intertwined processes, with inflammation exacerbating endothelial dysfunction and vice versa. The cross-talk between inflammatory cells, cytokines, and the endothelium contributes to a vicious cycle that perpetuates the prothrombotic environment. Disruption of endothelial homeostasis and the prothrombotic state may ultimately result in the formation of venous thrombi and the manifestation of VTE [27]. Understanding the impact of inflammation and endothelial dysfunction in OSA and VTE is crucial for developing targeted therapeutic interventions. Modulating inflammation and restoring endothelial function may hold promise for preventing and managing VTE in individuals with OSA. Future research aimed at elucidating the specific molecular mechanisms underlying these processes and exploring novel therapeutic strategies targeting inflammation and endothelial dysfunction may lead to improved outcomes in patients with both OSA and VTE.

Role of Sympathetic Activation and Hemodynamic Changes

The role of sympathetic activation and hemodynamic changes in the association between OSA and VTE is a significant aspect to consider. OSA is associated with sympathetic activation, which refers to the increased activity of the sympathetic nervous system. This is characterized by heightened sympathetic nerve activity and elevated levels of catecholamines such as adrenaline and noradrenaline [28].

Sympathetic activation profoundly affects the cardiovascular system, manifesting as heightened blood pressure, heart rate, and vascular tone. These physiological responses are integral to the regulation of circulatory dynamics. The interplay between sympathetic activity and VTE development is multifaceted. The elevation in blood pressure and heart rate initiated by sympathetic activation may contribute to an intricate interplay within the coagulation cascade, thereby influencing VTE risk. Concurrently, this heightened cardiovascular response can impact blood flow dynamics, potentially disrupting the normal hemodynamic equilibrium [29].

Remarkably, it is noteworthy that an increased sympathetic activity could potentially play a dual role in preventing VTE. By elevating blood pressure and augmenting vascular tone, sympathetic activation may discourage the formation of stagnant blood pools that are conducive to thrombus formation. In this context, the increased blood pressure and decreased blood stasis resulting from sympathetic activity might contribute to mitigating VTE risk. The intricate relationship between sympathetic activation, cardiovascular dynamics, and VTE warrants comprehensive investigation. Further research is essential to elucidate the precise mechanisms through which sympathetic activity impacts the coagulation system and blood flow patterns, thereby shedding light on its potential role in the prevention and pathogenesis of VTE [29].

Furthermore, the hemodynamic changes associated with sympathetic activation and the intermittent hypoxia experienced during apnea episodes in OSA can have additional consequences. The role of beta 2 agonists in this context warrants consideration. Beta 2 agonists, often used for bronchodilation in respiratory conditions, can influence sympathetic activity and vascular tone. In the setting of OSA, where sympathetic activity is already heightened, the interaction between beta 2 agonists and the sympathetic nervous system could potentially exacerbate hemodynamic changes. Intermittent hypoxia, a hallmark of OSA, refers to the recurring periods of oxygen deprivation during apnea events. These oxygen fluctuations can induce oxidative stress and inflammation, further contributing to the prothrombotic state [30]. This intricate interplay between sympathetic activation, beta 2 agonists, intermittent hypoxia, oxidative stress, and prothrombotic mechanisms requires careful examination to elucidate their combined effects on OSA-related cardiovascular complications.

The combination of sympathetic activation, hemodynamic changes, and intermittent hypoxia can lead to venous stasis and impaired venous return. Venous stasis refers to the slowed or stagnant blood flow within the veins, while impaired venous return refers to the reduced ability of blood to return to the heart efficiently. These factors create an environment predisposing individuals with OSA to venous thrombosis, potentially increasing the risk of VTE events [31]. Understanding the role of sympathetic activation and hemodynamic changes in the context of OSA and VTE provides insights into the underlying mechanisms and pathophysiology of the association. It highlights the importance of addressing sympathetic overactivity and managing hemodynamic alterations as part of the overall management strategies for individuals with OSA and their potential implications for VTE prevention.

Influence of Obesity and Metabolic Dysregulation

Obesity, characterized by excessive accumulation of body fat, is a well-established risk factor for OSA and VTE. In the context of OSA and VTE, obesity is closely linked to metabolic dysregulation, which plays a significant role in the development and progression of these conditions [32].

Adipose tissue, particularly visceral fat, is an inert energy storage depot and an active endocrine organ. It secretes various bioactive substances, including inflammatory cytokines (such as interleukin-6 and tumor necrosis factor-alpha) and adipokines (such as leptin and adiponectin). In obesity, the adipose tissue becomes dysfunctional, and releases increased pro-inflammatory mediators. This chronic low-grade inflammation contributes to a prothrombotic state and endothelial dysfunction, both of which are implicated in the pathogenesis of VTE [33].

Furthermore, obesity is associated with metabolic disturbances such as insulin resistance, dyslipidemia, and altered coagulation factors. Insulin resistance, a hallmark of obesity, leads to impaired glucose metabolism and increased circulating insulin levels. Insulin resistance and hyperinsulinemia promote a prothrombotic state by stimulating platelet activation and aggregation, impairing fibrinolysis, and promoting endothelial dysfunction. Dyslipidemia, characterized by elevated levels of triglycerides, low-density lipoprotein cholesterol, and decreased levels of high-density lipoprotein cholesterol, contributes to the development of atherosclerosis and subsequent VTE risk. Altered coagulation factors, including increased levels of fibrinogen and factor VIII, further enhance the thrombotic tendency in obese individuals [34].

The influence of obesity and metabolic dysregulation on the association between OSA and VTE is substantial. Obesity increases the risk of developing both OSA and VTE and exacerbates the pathophysiological mechanisms underlying these conditions. Targeting obesity through lifestyle modifications, including weight loss and improved metabolic health, may have a beneficial impact on reducing the risk and severity of both OSA and VTE. Additionally, addressing obesity-related metabolic abnormalities can improve overall cardiovascular health and reduce the prothrombotic state associated with VTE [35]. Understanding the intricate interplay between obesity, metabolic dysregulation, OSA, and VTE is crucial for developing comprehensive management strategies encompassing weight management, metabolic control, and preventing VTE events. By addressing obesity-related factors, clinicians can potentially improve outcomes and mitigate the adverse effects of OSA and VTE in affected individuals.

Clinical implications and management considerations

Screening and Identification of OSA in VTE Patients

Given the emerging association between OSA and VTE, it is crucial to prioritize the screening and identification of OSA in patients with VTE. Healthcare providers should be vigilant and recognize the potential coexistence of these conditions, understanding that addressing OSA in VTE patients may have significant clinical implications [2]. When evaluating patients with VTE, healthcare providers should consider the possibility of underlying OSA, particularly in individuals who share common risk factors such as obesity, older age, and comorbidities. Recognizing these shared risk factors can prompt clinicians to initiate a systematic approach to OSA screening in VTE patients [36].

Smartwatches have revolutionized health monitoring, capturing a wide spectrum of health data that holds potential for sleep apnea detection. The integration of artificial intelligence and advanced technology in these devices presents an evolving landscape worth exploring. In this context, it is noteworthy to consider recent advancements and updates in smartwatch capabilities regarding sleep apnea detection. These devices, equipped with various sensors, can monitor sleep patterns and associated physiological parameters. Incorporating such information could enhance the early identification of sleep apnea, leading to timely interventions and improved patient outcomes [37].

Home sleep apnea testing (HSAT) is another valuable tool that can be employed in the screening process. HSAT allows assessing respiratory parameters during sleep in the comfort of the patient's home. It involves using portable devices that monitor parameters such as airflow, oxygen saturation, and respiratory effort. HSAT provides a convenient and cost-effective alternative to laboratory-based polysomnography, the gold standard diagnostic test for OSA, and can aid in identifying individuals who may require further evaluation and treatment [38].

By implementing a systematic approach to OSA screening in VTE patients, healthcare providers can identify individuals who may benefit from further evaluation and management. Timely recognition and appropriate treatment of OSA in this population can potentially reduce the risk of VTE recurrence, improve overall outcomes, and optimize patient care [39]. It is important to note that the screening and identification of OSA in VTE patients should be performed in a multidisciplinary setting involving collaboration between sleep medicine specialists, pulmonologists, cardiologists, and other relevant healthcare providers. This collaborative approach ensures comprehensive evaluation and management of both conditions, leading to more effective and personalized patient care with the coexistence of OSA and VTE.

Implications for Treatment and Management Strategies

OSA in individuals with VTE has important implications for their treatment and management strategies. Recognizing and addressing OSA in VTE patients is crucial for achieving optimal patient outcomes [40].

Treating OSA in individuals with VTE can lead to various benefits. First and foremost, managing OSA can alleviate the symptoms associated with the condition such as loud snoring, daytime sleepiness, and disrupted sleep patterns. This improvement in sleep quality can positively impact the overall well-being of the patient [41].

Furthermore, addressing OSA in VTE patients may reduce the risk of recurrent VTE events. OSA is characterized by repetitive episodes of partial or complete upper airway obstruction during sleep, leading to intermittent hypoxia and oxidative stress. These physiological changes can promote a prothrombotic state and contribute to the development or exacerbation of VTE. By effectively treating OSA, the frequency and severity of these episodes can be reduced, potentially lowering the risk of VTE recurrence [24].

In addition to reducing the risk of VTE, managing OSA-related factors can have broader implications for overall health and VTE-related complications. For instance, interventions that target intermittent hypoxia and inflammation associated with OSA may positively impact endothelial function, inflammation markers, and the balance of coagulation and fibrinolysis. This, in turn, may help mitigate VTE-related complications such as post-thrombotic syndrome and chronic thromboembolic pulmonary hypertension [42].

The treatment and management strategies for individuals with OSA and VTE typically involve a multidisciplinary approach. Healthcare providers specializing in sleep medicine, pulmonology, cardiology, and hematology collaborate to develop personalized treatment plans. Continuous positive airway pressure (CPAP) therapy is often a primary treatment modality for OSA, as it helps maintain an open upper airway during sleep. Lifestyle modifications, including weight loss, regular exercise, and avoidance of sedatives and alcohol, can also significantly manage OSA and reduce VTE risk [43].

Role of CPAP Therapy

CPAP therapy is considered the gold standard treatment for OSA. It involves using a device that delivers a continuous flow of pressurized air through a mask worn over the nose and mouth during sleep. The positive airway pressure acts as a pneumatic splint, preventing the collapse of the upper airway and maintaining an open and unobstructed breathing passage [44].

CPAP therapy has demonstrated potential benefits in the context of VTE and coexisting OSA. By maintaining an open airway throughout the night, CPAP therapy can improve nocturnal oxygenation levels, reduce the frequency and severity of apnea and hypopnea events, and alleviate the episodes of intermittent hypoxia typically experienced by individuals with OSA [5].

One of the mechanisms through which CPAP therapy may impact the association between OSA and VTE is addressing systemic inflammation and endothelial dysfunction. OSA is associated with chronic low-grade inflammation and endothelial dysfunction, contributing to a prothrombotic state. CPAP therapy has been shown to reduce systemic inflammatory markers and improve endothelial function, potentially attenuating the prothrombotic effects associated with OSA. These positive effects on inflammation and endothelial function may have implications for reducing the risk of VTE events and improving overall cardiovascular health in individuals with coexisting OSA and VTE [45]. While CPAP therapy is the primary treatment modality for OSA, its effectiveness in the context of VTE outcomes is an area of ongoing research. Randomized controlled trials (RCTs) and prospective studies are needed to evaluate further the impact of CPAP therapy on VTE-related outcomes, such as the occurrence and recurrence of VTE events, and its potential role in improving long-term prognosis.

Potential Benefits of Lifestyle Modifications and Weight Loss

Lifestyle modifications, such as weight loss, exercise, and healthy dietary practices, play a crucial role in the management of both OSA and VTE. Among these lifestyle changes, weight loss holds particular importance, especially in individuals with obesity who are at higher risk for both OSA and VTE. Losing weight can significantly improve OSA severity by reducing upper airway obstruction during sleep and promoting better breathing patterns. Furthermore, weight loss has been associated with a reduced risk of developing VTE, as obesity is a known risk factor for thromboembolic events [46].

Regular physical activity and exercise are also beneficial in managing OSA and VTE. Engaging in regular exercise not only promotes cardiovascular health but also aids in weight management, which can have a positive impact on both conditions. Exercise has been shown to improve OSA symptoms, increase exercise tolerance, and reduce the severity of sleep apnea. Additionally, physical activity improves blood circulation and venous return, potentially reducing the risk of VTE occurrence or recurrence [47].

Adopting healthy dietary practices is another important component of lifestyle modifications. Consuming a balanced diet that includes a variety of nutrient-rich foods is essential for overall health and can positively influence both OSA and VTE outcomes. Reducing sodium intake is particularly important for individuals with OSA, as excessive sodium consumption can contribute to fluid retention, leading to increased upper airway narrowing and worsening of OSA symptoms. A well-balanced diet that includes fruits, vegetables, whole grains, lean proteins, and healthy fats can provide essential nutrients and support overall cardiovascular health, potentially benefiting individuals with both OSA and VTE [48].

Pharmacological Interventions and Anticoagulant Therapy

Pharmacological interventions and anticoagulant therapy are crucial in managing VTE. While these interventions are essential components of VTE treatment, their specific impact on the association between OSA and VTE is still an area of ongoing research [49].

Anticoagulant therapy, such as anticoagulant medications like heparin and warfarin, remains the cornerstone for treating and preventing VTE. The decision to initiate anticoagulation is based on the severity and clinical presentation of the VTE event, and the presence of OSA does not directly influence it. Anticoagulation aims to prevent the progression of existing blood clots, reduce the risk of recurrent VTE, and mitigate the potential complications associated with VTE [50].

However, healthcare providers should consider potential drug interactions or modifications in anticoagulant therapy when managing patients with both OSA and VTE. OSA-specific interventions, such as CPAP therapy, may impact the efficacy or safety of anticoagulant medications. For example, CPAP therapy may improve oxygenation and alleviate respiratory disturbances in OSA patients, potentially affecting the stability of anticoagulant dosing or the risk of bleeding. Therefore, close monitoring and adjustment of anticoagulant therapy may be necessary for individuals receiving CPAP therapy and anticoagulation [51].

In addition to anticoagulant therapy, other pharmacological interventions may be considered in managing VTE. For instance, thrombolytic agents may be used in cases of massive or high-risk VTE to dissolve blood clots more rapidly. However, using these agents in individuals with coexisting OSA requires careful consideration due to potential risks and contraindications [52]. Further research is needed to understand better the effects of specific pharmacological interventions on the OSA-VTE association. Future studies should investigate the optimal management strategies and potential modifications in pharmacotherapy for individuals with both OSA and VTE, considering this patient population's unique characteristics and needs.

Future directions and research gaps

Areas for Further Research and Investigation

While significant progress has been made in understanding the emerging association between OSA and VTE, several areas still require further research and investigation. These areas include:

Long-term outcomes: Longitudinal studies with extended follow-up periods are necessary to assess the long-term effects of OSA on VTE recurrence, mortality, and overall prognosis. Understanding the impact of OSA on VTE outcomes over an extended period is crucial for determining the optimal management strategies and interventions.

Underlying mechanisms: More research is needed to elucidate the specific molecular and cellular pathways that link OSA and VTE. Key areas of focus include investigating the role of intermittent hypoxia, oxidative stress, inflammation, and endothelial dysfunction in the development and progression of VTE in individuals with OSA. Understanding these mechanisms can help identify potential therapeutic targets for preventing or mitigating VTE in OSA patients.

Identifying high-risk individuals: Identifying biomarkers or clinical predictors that accurately identify individuals at high risk of developing OSA and VTE is essential for early detection and intervention. Such identification would enable targeted screening and proactive management strategies to mitigate the risk of VTE in individuals with OSA.

Impact of treatment on VTE outcomes: Studies are needed to evaluate the effect of OSA treatment, specifically CPAP therapy, on VTE outcomes and recurrence rates. Assessing the potential benefits of managing OSA in VTE patients through interventions like CPAP therapy can provide insights into the effectiveness of OSA treatment in reducing VTE-related complications and improving patient outcomes.

Prospects for RCTs

RCTs are considered the gold standard for evaluating the effectiveness of interventions. Future RCTs focusing on managing OSA in VTE patients are essential to determine the efficacy of different treatment modalities, compare different interventions, and assess their impact on VTE-related outcomes. RCTs can provide more robust evidence regarding the benefits of OSA treatment in reducing VTE recurrence and improving overall patient outcomes.

Development of Targeted Interventions and Personalized Medicine

The field of personalized medicine holds promise for managing OSA and VTE. As our understanding of the underlying mechanisms and individual variability increases, developing targeted interventions tailored to specific patient profiles becomes crucial. Personalized approaches may involve identifying subgroups of patients most likely to benefit from OSA treatment, optimizing treatment strategies based on individual characteristics, and considering genetic and biomarker-based approaches to tailor interventions.

Importance of Multidisciplinary Collaboration and Shared Guidelines

Given the complex interplay between OSA and VTE, multidisciplinary collaboration is essential. Close cooperation between sleep medicine specialists, pulmonologists, cardiologists, hematologists, and other relevant healthcare providers can facilitate a comprehensive approach to managing patients with both conditions. Collaboration can lead to the development of shared guidelines that incorporate recommendations for the screening, diagnosis, treatment, and follow-up of individuals with coexisting OSA and VTE, ensuring standardized and evidence-based care.

## Conclusions

In conclusion, the emerging association between OSA and VTE represents a significant area of research and clinical interest. OSA, characterized by recurrent upper airway obstruction during sleep, and VTE, encompassing DVT and PE, are both complex conditions with multifactorial etiologies. The evidence gathered thus far supports a potential bidirectional relationship between OSA and VTE, wherein the presence of one condition may increase the risk or exacerbate the severity of the other. The review article provided an in-depth exploration of the background, pathophysiology, clinical presentation, and impact of both OSA and VTE. Shared risk factors, such as obesity, older age, and comorbidities, likely contribute to the observed association between the two conditions. Mechanistic pathways linking OSA and VTE include intermittent hypoxia, oxidative stress, inflammation, endothelial dysfunction, sympathetic activation, and metabolic dysregulation. Clinical studies, including epidemiological investigations, prospective studies, and mechanistic research, have provided valuable insights into the OSA-VTE association, although further research is still needed to fully elucidate the underlying mechanisms and long-term outcomes. The clinical implications of the OSA-VTE association are significant. Screening and identification of OSA in VTE patients, along with appropriate treatment and management strategies, can potentially improve patient outcomes and reduce the risk of recurrent VTE events. CPAP therapy, lifestyle modifications, weight loss, and personalized medicine approaches hold promise in managing both OSA and VTE. Additionally, multidisciplinary collaboration and the development of shared guidelines are vital for providing standardized and evidence-based care to individuals with coexisting OSA and VTE.
